# Osmolarity regulates *Caenorhabditis elegans* egg-laying behavior via chemosensory and biophysical mechanisms

**DOI:** 10.1242/jeb.250132

**Published:** 2025-11-07

**Authors:** Emmanuel Medrano, Karen Jendrick, Julian McQuirter, Claire Moxham, Dominique Rajic, Lila Rosendorf, Liraz Stilman, Dontrel Wilright, Kevin M. Collins

**Affiliations:** Department of Biology, University of Miami, 1301 Memorial Drive, Coral Gables, FL 33146, USA

**Keywords:** Calcium, Hydrostatic pressure, Neurotransmitter, Vulval muscle, Circuit, HSN command neuron

## Abstract

Animals alter their behavior in response to changes in the environment. Upon encountering hyperosmotic conditions, the nematode worm *Caenorhabditis elegans* initiates avoidance and cessation of egg-laying behavior. While the sensory pathway for osmotic avoidance is well understood, less is known about how egg laying is inhibited. We analyzed egg-laying behavior after acute and chronic shifts to and from hyperosmotic media. Animals on 400 mmol l^−1^ sorbitol stop laying eggs immediately but then resume ∼3 h later, after accumulating additional eggs in the uterus. Surprisingly, the hyperosmotic cessation of egg laying still occurred in known osmotic avoidance signaling mutants. Acute hyperosmotic shifts in hyperosmotic-resistant mutants overproducing glycerol also blocked egg laying, but these animals resumed egg laying more quickly than similarly treated wild-type animals. These results suggest that hyperosmotic conditions disrupt a ‘high-inside’ hydrostatic pressure gradient required for egg laying. Consistent with this hypothesis, animals adapted to hyperosmotic conditions laid more eggs after acute shifts back to normosmic conditions. Optogenetic stimulation of the HSN egg-laying command neurons in hyper-osmotic treated animals led to fewer and slower egg-laying events, an effect not seen following direct optogenetic stimulation of the postsynaptic vulval muscles. Hyperosmotic conditions also affected egg-laying circuit activity with the vulval muscles, showing reduced Ca^2+^ transient amplitudes and frequency even after egg-laying resumes. Together, these results indicate that hyperosmotic conditions regulate egg-laying via two mechanisms: a sensory pathway that acts to reduce HSN excitability and neurotransmitter release, and a biophysical mechanism where a hydrostatic pressure gradient reports egg accumulation in the uterus.

## Teaser

**Summary:** Hyperosmotic conditions inhibit *Caenorhabditis elegans* egg laying through both a sensory pathway and a biophysical pathway affecting a ‘high-inside’ hydrostatic pressure gradient.

## INTRODUCTION

Like many other animals, *Caenorhabditis elegans* regulates behaviors such as locomotion and egg laying in response to external environmental conditions (Sawin, 1996; Sawin et al., 2000; Yu et al., 2017; Zhu et al., 2014). Shifts to low external osmolarity promote *C. elegans* egg laying (Zhang et al., 2008, 2010), while high external osmolarity inhibits it (Sawin, 1996). How changes in external osmolarity mediate these effects remains unclear. Osmolarity could influence egg laying through two broad types of mechanisms: (1) a sensory pathway, in which neurons detect osmotic changes and signal to the egg-laying circuit, and (2) a biophysical pathway, in which osmotic shifts directly change the worm's internal hydrostatic pressure and thereby alter physical egg expulsion. Below, we review evidence for each of these possibilities.

Genetic screens for *C. elegans* mutants that affect osmotic avoidance have identified numerous genes required for the development and function of chemosensory neurons that mediate osmotic avoidance (Culotti and Russell, 1978). Nociceptive sensory neurons signal to inhibit egg laying in response to unfavorable environmental conditions, and thus external osmolarity may similarly function through a sensory pathway to regulate egg-laying behavior. For example, elevated CO_2_ activates the BAG nociceptive neurons, driving the release of neuropeptides which bind to the EGL-6 receptor expressed on the HSN command motor neurons which control egg laying (Fenk and de Bono, 2015; Ringstad and Horvitz, 2008). EGL-6 couples to Gα_o_ which signals through downstream effectors including IRK-1 to stabilize HSN electrical excitability (Emtage et al., 2012; Ravi et al., 2021), reducing serotonin release and vulval muscle contractility (Collins et al., 2016). However, it is unknown whether the observed regulation of egg laying by external osmolarity is mediated through similar cellular and molecular mechanisms.

Hyperosmotic inhibition of egg laying may also be mediated by a sensory-independent or biophysical pathway. Normally, under hyperosmotic stress, *C. elegans* shows a rapid (within minutes) decrease in relative body volume due to water loss to the surrounding environment. Genetics screens for mutant animals resistant to changes in osmolarity (Osr) have identified pathways that function internally to prevent the loss of water and hydrostatic pressure (Solomon et al., 2004; Wheeler and Thomas, 2006). For example, *osr-1* mutants display normal viability, size and motility when grown in hyperosmotic environments (Solomon et al., 2004). Acclimatization to osmotic stress is accomplished by the accumulation of glycerol, which leads to an increase in relative body volume (Lamitina et al., 2004). *Caenorhabditis elegans* expresses various aquaporins that have been shown to increase water or glycerol permeability when exogenously expressed in *Xenopus* oocytes. Worms maintain their internal pressure hydrostatically and are normally highly pressurized (∼140 kPa). Puncturing their cuticle with a fine needle leads to expulsion of internal organs (Gilpin et al., 2015; Avery and Thomas, 1997; Harris and Crofton, 1957). As such, osmotic regulation of egg laying may be mediated by the changes in internal pressure that accompany exposure to hyperosmotic or hypoosmotic environments. These changes, in turn, regulate the ability of hydrostatic pressure to drive release of eggs into the environment following vulval muscle contraction.

We have previously shown that changes in internal stretch and pressure that accompany egg accumulation affect egg-laying circuit activity and behavior. Genetically or chemically sterilized animals lacking eggs have reduced Ca^2+^ activity in the HSN command neurons and the postsynaptic vulval muscles (Ravi et al., 2018b). Conversely, artificial induction of egg accumulation drives a homeostatic increase in HSN Ca^2+^ activity that drives the release of additional eggs (Ravi et al., 2018b). Feedback of egg accumulation can modulate the egg-laying vulval muscles independent of the HSNs (Collins et al., 2016; Ravi et al., 2021), suggesting the vulval muscles themselves are the ultimate target. Consistent with this, acute injection to mimic the stretch-dependent feedback of egg accumulation is sufficient to induce egg-laying circuit activity, with the vulval muscles among the first to respond (Medrano and Collins, 2023). This suggests that changes in hydrostatic pressure resulting from changes in egg accumulation or osmolarity may facilitate changes in egg laying. Within a stretch model, hypoosmotic activation of egg laying has the same physical consequences as acute injection. Acute shifts of animals to hypoosmotic conditions drives a rapid influx of water into animals (Lamitina et al., 2004). The presumable increase in hydrostatic pressure inside the animal then promotes circuit activity and egg laying. Conversely, a decrease in internal hydrostatic pressure would be predicted to inhibit egg-laying circuit activity and behavior, as previously observed (Zhang et al., 2008, 2010).

Here, we used manipulations of osmolarity to understand how sensory input and the hydrostatic pressure gradient control egg laying. Our results suggest a hyperosmotic environment regulates egg-laying behavior through both biophysical and chemosensory pathways.

## MATERIALS AND METHODS

### Nematode strains and culture

*Caenorhabditis elegans* strains were maintained as hermaphrodites at 20°C on nematode growth medium (NGM) agar plates with *E. coli* OP50 as a source of food as described previously (Brenner, 1974). All strains are derived from the Bristol N2 wild-type strain. Behavior assays were performed using age-matched adult hermaphrodites ∼24–30 h past the late L4 stage using recommended sample sizes (Chase and Koelle, 2004).

A list of strains, mutants and transgenes used in this study can be found in Table 1. The *tax-2; osm-8* double mutant was built as follows. N2 males were crossed into PR694 *tax-2(p694) I* hermaphrodites, and the *tax-2/+* cross-progeny males were then crossed to MT3571 *osm-8(n1518) II* hermaphrodites. *tax-2/+; +/osm-8* heterozygous mutants were identified using PCR-based genotyping. *p694* was identified by duplex PCR using the following oligonucleotides: tax-2(p694)-mut-fwd: TGA TGA CTG CTT GGC AAC GGA CTT; tax-2(p694)-wt-fwd: GAT AGA CAG GTA CAT AAT CTT CAG AAT CTG; and tax-2(p694)-rev: TGC AGA AAT GCT CGA AGT AGC CCA. The premature TAG stop codon of *n1518* was identified using a tetra-ARMS based approach (Collins and Ke, 2012; Ye et al., 2001) using the following oligonucleotides osm-8(n1518)-fwd: GCA GAT GCG CCA ACA CTC AAG GTG; osm-8(n1518)-rev: GCC ACG GTT CAA TAT AGG GTT TAA ACA GCC G; osm-8(n1518)-TAG-fwd-inner A: CAT CCA TTA AGT ACT TCA AGA AGC TTC TA; osm-8(n1518)-TAG-rev inner G: GTC TCT GTA GTC GTT GAA ACA TTT ACC. Homozygous *tax-2; osm-8* animals were kept as strain MIA623.

**Table 1.** Strains used in this study

Strain	Genotype and description	Source
N2	Wild-type	Brenner (1974)
CB1387	*daf-10(e1387) IV* [amphid/phasmid development defect]	CGC
CG21	*egl-30(tg26) I; him-5(e1490) V* [gain-of-function G_q_ signaling mutant; hyperactive for egg laying]	CGC
CX10	*osm-9(ky10) IV* [TRPV channel mutant]	CGC
CX2205	*odr-3(n2150) V* [Gα protein involved in chemosensation]	CGC
CX4544	*ocr-2(ak47) IV* [TRPV channel mutant]	CGC
DG1856	*goa-1(sa734) I* [G_o_ signaling mutant hyperactive for egg laying]	CGC
DR86	*daf-19(m86) II* [sensory cilium formation mutant]	CGC
IB16	*ceh-17(np1) I* [axon guidance mutant in sensory neurons]	CGC
JT204	*daf-12(sa204) X* [defective dauer formation mutant used to suppress high dauer incidence in *daf-19* mutants]	CGC
JT6924	*daf-19(m86) II; daf-12(sa204) X* [double mutant for sensory cilium formation and defective dauer formation]	CGC
LX671	*ocr-2(vs29) IV* [dominant-negative TRPV channel mutant; hyperactive for egg laying]	CGC
LX1836	*wzIs30 IV; lite-1(ce314) lin-15(n765ts) X* [HSN Channelrhodopsin]	Collins et al. (2016)
LX1918	*vsIs164 lite-1(ce314) lin-15(n765ts) X* [vulval muscles expressing GCaMP5, mCherry]	Collins et al. (2016)
LX1932	*wzIs30 IV; lite-1(ce314) vsIs164 lin-15(n765ts) X* [HSN Channelrhodopsin; vulval muscle GCaMP5, mCherry]	Collins et al. (2016)
MIA229	*keyIs48; lite-1(ce314) X lin-15(n765ts) X* [vulval muscle Channelrhodopsin-2]	Kopchock et al. (2021)
MIA250	*keyIs48; vsIs164 lite-1(ce314) X lin-15(n765ts) X* [vulval muscle Channelrhodopsin-2; vulval muscle GCaMP5, mCherry]	Kopchock et al. (2021)
MIA623	*tax-2(p694) I; osm-8(n1518) II*	This study
MT3564	*osm-7(n1515) III* [osmotic avoidance mutant, constitutive production of glycerol]	CGC
MT3571	*osm-8(n1518) II* [osmotic avoidance mutant, constitutive production of glycerol]	CGC
MT3641	*osm-10(n1602) III* [osmotic avoidance response mutant]	CGC
PR678	*tax-4(p678) III* [cyclic nucleotide-gated channel subunit; defective in chemotaxis]	CGC
PR694	*tax-2(p694) I* [cyclic nucleotide-gated channel subunit; defective in chemotaxis to everything except pyridine]	CGC

### High osmolarity plates

Two different sets of high osmolarity plates were used in all experiments: 1.7% agar alone and NGM agar supplemented with sorbitol. Agar plates were made as described previously (Alkema et al., 2005), adding acetic acid to a final concentration of 2 mmol l^−1^. Prior to the agar solidifying, sorbitol was added from a 4 mol l^−1^ stock to either 100 mmol l^−1^ or 400 mmol l^−1^ final concentration. For long-term growth and shift experiments (see below), NGM media contained 100 mmol l^−1^ or 400 mmol l^−1^ sorbitol added from a 4 mol l^−1^ stock solution. For the optogenetic experiments (see below), NGM at ∼400 mOsm was prepared by adding sorbitol to 350 mmol l^−1^ final concentration from a 4 mol l^−1^ stock. NGM alone (∼50 mOsm) was used as the ‘control’ media for these experiments. Agar-only or NGM-agar media were then poured into 10–60 mm plates and allowed to dry for at least 1 day. All plates were seeded with OP50 bacteria. To allow for sufficient growth of bacteria, OP50 bacteria were concentrated 10 times through centrifugation. Plates were then seeded with 25 µl of 10× concentrated OP50 bacteria. To discourage animals from crawling up the sides of the plate where they would otherwise desiccate, 25 µl of 50 mmol l^−1^ copper sulfate was used to form a ring on the edge of plates used in egg-laying behavior assays. We observed no changes in egg-laying behavior on OP50-seeded plates with or without the ring of copper sulfate. This was true for both wild-type and *tax-2(p694)* mutant animals on either 100 or 400 mmol l^−1^ sorbitol plates (data not shown).

### Egg laying in high osmolarity

**Fig. 1.**
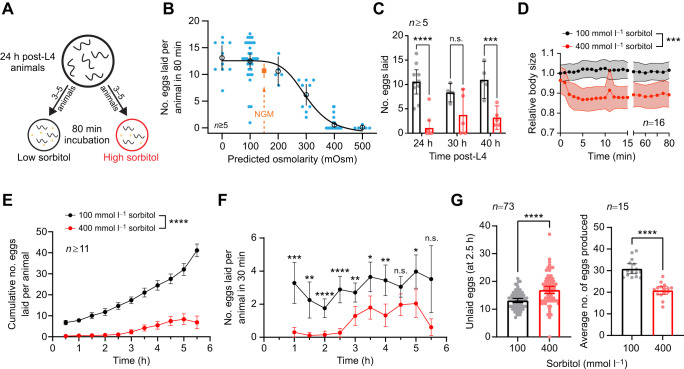
**Hyperosmotic inhibition of *Caenorhabditis elegans* egg-laying behavior is restored after chronic exposure.** (A) Diagram showing the assay for assessing egg laying in hyperosmotic conditions. Animals were placed onto agar plates containing different concentrations of sorbitol, from low (0 mmol l^−1^) to high (500 mmol l^−1^). After an 80 min period, the number of eggs laid on the plate was counted. (B) Dose–response curve showing the mean number of eggs laid after 80 min by animals on plates with different predicted osmolar concentrations. Open black circles indicate the mean number of eggs laid per condition (0–500 mmol l^−1^ sorbitol), and solid blue circles indicate the mean number of eggs laid per animal (3–5 animals per plate; *n*≥5 replicates per condition). The solid black line indicates the fitted four-parameter logistic curve with an estimated IC_50_ of 302 mmol l^−1^ sorbitol (adjusted *R*^2^=0.7936; Hill slope=−6.796). For comparison, the orange square shows the average egg-laying response of animals placed on nematode growth medium (NGM) plates with an approximate osmolarity of 150 mOsm. These NGM data were not included in the curve fit. (C) Scatterplot with bar graphs showing the mean number of eggs laid by 24, 30 or 40 h post-L4 animals on 100 mmol l^−1^ (black) or 400 mmol l^−1^ (red) sorbitol plates. Asterisks indicate significance (****P<0.0001, ***P<0.001; n.s., not significant; two-way ANOVA with Bonferroni correction for multiple comparisons). (D) Relative body size over time of animals placed acutely onto 100 or 400 mmol l^−1^ sorbitol plates (***P<0.001; unpaired *t*-test). (E) Cumulative number of eggs laid over time by animals on sorbitol plates (****P<0.0001; mixed-effects model with Bonferroni's correction for multiple comparisons). (F) Egg-laying rate (number of eggs laid per each 30 min interval) from data in E (****P<0.0001, ***P<0.001, **P<0.01, *P<0.05; n.s., not significant; mixed-effects model with Bonferroni's correction for multiple comparisons). (G) Left: bar graph with scatterplot showing the number of unlaid eggs within the uterus of animals exposed to 100 or 400 mmol l^−1^ sorbitol for a period of 2.5 h (left). Right: average number of eggs produced by animals in either sorbitol condition, obtained by combining the number of laid and unlaid eggs. All dots in B–G (except for G left) represent the average response from multiple animals within a plate. The egg-laying response from a plate was divided by the number of animals on the plate to obtain the average response on a per animal basis. *N* is the number of plates analyzed per condition, each bearing 3–5 worms. Error bars represent ±95% confidence intervals for the mean.

To assess how high osmolarity affects egg-laying behavior, we placed 24 h post-L4 animals onto agar plates containing either 100 or 400 mmol l^−1^ sorbitol (Fig. 1A); 3–5 animals were placed on replicates of each plate and allowed to lay eggs for 80 min. After this time, the total number of eggs laid by all animals was counted for each condition. The average number of eggs laid per animal was calculated by dividing the total number of eggs laid on a plate by the number of animals on that plate (3–5). This same procedure was used in the candidate mutant screen to determine the effect of high osmolarity on the egg laying of these mutants. For chronic exposure experiments, laid eggs were counted every 30 min while animals were maintained on agar–sorbitol plates for up to 5.5 h. Unlaid eggs were quantified as described previously (Chase and Koelle, 2004).

### Relative body size estimate

To measure changes in relative body size after acute shifts to high osmolarity, 8 animals were placed onto 100 or 400 mmol l^−1^ sorbitol-supplemented agar plates. Animals were then recorded for 80 min using a FLIR Grasshopper 3 camera mounted on a stereomicroscope (Leica M165 FC). Recordings were then analyzed in ImageJ to measure total body surface area. Measurements were performed every minute for the first 15 min and then every 5 min thereafter. All measurements were normalized by averaging the area of all 8 animals during the first frame of the recording and subtracting this average from all other measurements to determine the normalized area.

### High osmolarity rescue experiments

To measure the effects of rescuing animals from high osmolarity on egg laying, animals were moved from 400 mmol l^−1^ to 100 mmol l^−1^ sorbitol plates. When animals were incubated for only 1 h, sorbitol–agar plates were used, but when animals were incubated on high osmolarity for one or more generations, the sorbitol-supplemented NGM agar plates were used (described above). This was done to prevent any defects in development or changes in behavior that may accompany growth in non-nutrient growth media.

### Optogenetics

MIA229 and LX1932 strains expressing Channelrhodopsin-2 (ChR2) in either the vulval muscles or HSNs were used in optogenetic assays (see Table 1). Prior to optogenetic exposure, plates containing all-trans retinal (ATR) (Sigma-Aldrich, R2500) were prepared by adding 200 µl of warmed OP50 bacteria in B Broth containing 0.4 mmol l^−1^ ATR. Animals were typically maintained on ATR plates for multiple generations. A set of control animals were grown on plates lacking ATR. Animals were picked to NGM agar alone or NGM agar supplemented with 350 mmol l^−1^ sorbitol (∼400 mOsm total) for 0.5–1 h prior to imaging. From these plates, an agar chunk was cut out and placed worms-side down onto a 25×60 mm glass coverslip for imaging, as previously described (Collins and Koelle, 2013; Ravi et al., 2018a). Worms were imaged through a ×20 objective on a Zeiss Axio Observer.Z1 inverted compound microscope. Animals were exposed to a light off–on–off sequence that lasted 90 s. ChR2 was excited continuously at the 30 s time point for a 30 s duration by a 470 nm LED. An OTPG4 TTL Pulse Generator (Doric Instruments) was used to trigger a Grasshopper 3 camera (FLIR) which captured 2×2 binned, 1024×1024 8-bit JPEG brightfield image sequences of behavior at 75 frames s^−1^. Egg laying was recorded during the whole 90 s recording. Measurement of time until the first egg-laying event and duration of physical egg expulsion was performed in ImageJ from brightfield recordings by counting the number of frames required for each event to occur. The duration of egg expulsion was measured from the moment the egg surface emerged just past the vulval opening until it was completely released into the environment.

### Vulval muscle Ca^2+^ imaging

Calcium imaging was performed in freely behaving LX1918, LX1932 and MIA250 animals (see Table 1) using a Zeiss Axio Observer microscope at ×20 magnification, as previously described (Collins et al., 2016; Kopchock et al., 2021; Ravi et al., 2018a). Animals were exposed to 100 or 400 mmol l^−1^ sorbitol-supplemented agar plates for a minimum of 60 min up to a maximum of 4.5 h prior to Ca^2+^ imaging. Data were collected from animals alternating between treatment conditions (e.g. one animal from 100 mmol l^−1^ sorbitol followed by the next from 400 mmol l^−1^ sorbitol). Non-optogenetic LX1918 recordings (see below) were typically 10 min long with time of osmotic exposure noted for recorded individuals. Optogenetic recordings (see below) were typically 90 s with the same 470 nm blue light used to excite GCaMP5 fluorescence also serving to stimulate the co-expressed ChR2. A 10 ms on/40 ms off duty cycle was used to trigger 470 and 590 nm LED illumination (Zeiss Colibri.2) and excite ChR2 and GCaMP5/mCherry fluorescence, which was separated via a Hamamatsu Gemini W-View beam splitter and imaged by an ORCA Flash 4.0 V2 sCMOS camera at 20 frames s^−1^. Sample recordings from animals grown without ATR were collected on the same day to ensure ATR-plus animals were showing a robust optogenetic response. The timing of egg-laying events was measured from synchronously recorded brightfield behavior data. Data from at least three independent experiments each containing multiple animals from all treatments were pooled for subsequent analysis.

### Statistical procedures

Results from independent experiments over several days were pooled (unless otherwise noted). Statistical analyses were performed using Prism version 8-10 (GraphPad). Isogenic control animals were always tested alongside mutants to account for possible batch effects. Student's *t*-test was performed in experiments with two conditions or genotypes when the data were normally distributed. In cases in which multiple genotypes or conditions were compared, the data were analyzed by one-way or two-way ANOVA. In cases in which data were found to be non-normal, a non-parametric equivalence test was performed (e.g. Mann–Whitney or Kruskal–Wallis). All tests were corrected for multiple comparisons (Bonferroni for ANOVA, Dunn for Kruskal–Wallis).

## RESULTS

### *Caenorhabditis elegans* resumes egg-laying behavior after chronic exposure to hyperosmotic stress

To test osmotic inhibition of egg laying, we developed an assay in which animals were placed on solid media containing different concentrations of sorbitol. Sorbitol was chosen as a non-metabolic sugar that can induce the osmotic stress response in *C. elegans* (Chandler-Brown et al., 2015). After an 80 min incubation period, we then counted the number of eggs laid (Fig. 1A). We found a dose-dependent inhibition, with media containing ≥400 mmol l^−1^ sorbitol strongly blocking egg laying (Fig. 1B). Treatment with 100 mmol l^−1^ sorbitol, which approximates the osmolarity of NGM (∼150 mOsm) (Yu et al., 2017), led to a similar number of eggs laid as for animals on NGM. The inhibition on elevated sorbitol media was not dependent on age, as older animals also laid fewer eggs overall. This reduction was not significant in animals measured 30 h post-L4, but was in those measured at 40 h (Fig. 1C). Consistent with prior studies (Lamitina et al., 2004), we also saw a ∼15% decrease in relative body size immediately following exposure to 400 mmol l^−1^ sorbitol that persisted for at least 1 h (Fig. 1D).

While inhibition of egg laying via hyperosmotic exposure (Zhang et al., 2008) has been previously shown, we wondered whether *C. elegans* could acclimate and resume egg laying after chronic exposure. To do this, we recorded egg laying every 30 min for a 5.5 h period by animals placed on media containing either 100 or 400 mmol l^−1^ sorbitol. While egg laying was initially blocked on 400 mmol l^−1^ sorbitol, animals resumed egg laying after ∼2.5 h **(**Fig. 1E). This recovery was faster than the reported 24 h required for acclimation to hyperosmotic environments via accumulation of glycerol (Lamitina et al., 2004). Although egg laying resumed, the rate of egg laying was lower from 400 mmol l^−1^ than from 100 mmol l^−1^ sorbitol-treated animals during all 30 min intervals (Fig. 1F). Consequently, animals on 400 mmol l^−1^ sorbitol media retained 17 eggs on average, which was significantly more than the 13 retained by animals on 100 mmol l^−1^ sorbitol after 2.5 h of exposure (Fig. 1G, left). However, animals on 400 mmol l^−1^ sorbitol also showed reduced egg production when accounting for the total number of eggs laid on plates and those retained within the uterus (Fig. 1G, right). Together, these results show that hyperosmotic media temporarily inhibits egg laying in *C. elegans*. Egg laying resumes after 2.5 h of hyperosmotic exposure, correlating with an increase in egg accumulation within the uterus. This resumption of egg laying supports our previous work in which increased stretch from egg accumulation serves as an additional signal to promote egg-laying active states (Medrano and Collins, 2023; Ravi et al., 2018b).

### Hyperosmolarity inhibits egg laying in chemosensory osmotic avoidance but not osmoregulatory mutants

**Fig. 2.**
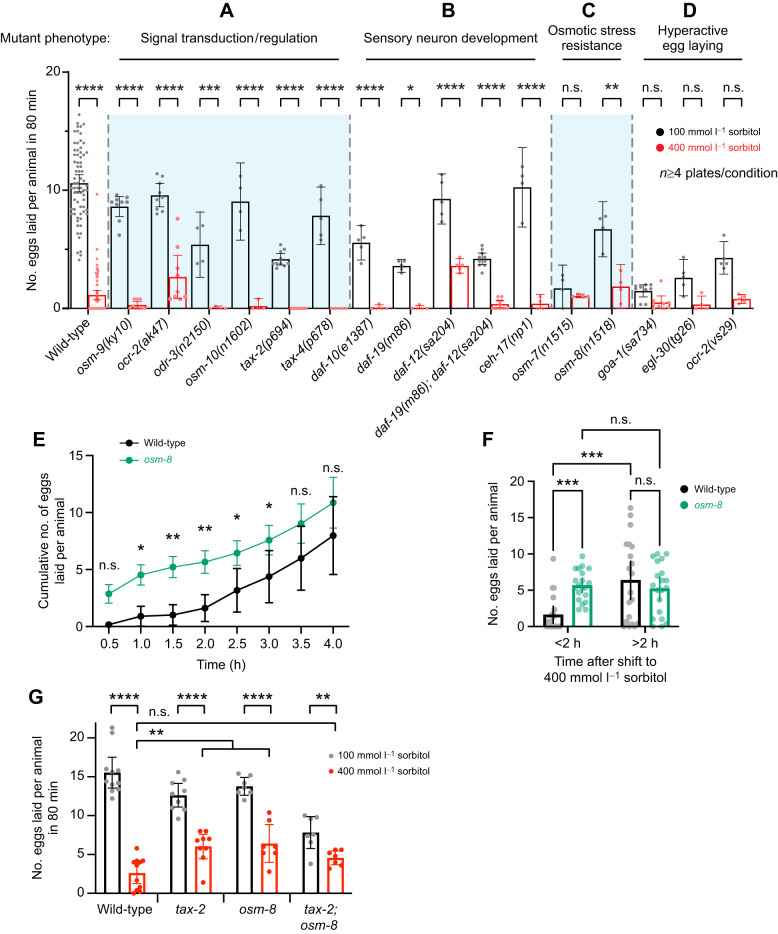
**Mutants defective in osmosensation, sensory neuron development, the osmotic stress response or hyperactive egg laying still show reduced egg laying in high osmolarity.** (A–D) Scatterplot showing the average number of eggs laid within 80 min by wild-type or mutant animals incubated on 100 or 400 mmol l^−1^ sorbitol plates. Mutants are separated by category labels at the top (A, signal transduction/regulation; B, sensory neuron development; C, osmotic stress resistance; D, hyperactive egg laying) and by the vertical dotted lines and shaded regions. Each dot represents the total number of eggs laid on one plate divided by the total number of animals on that plate. (E) Time course of cumulative egg laying of wild-type (black) or *osm-8(n1518)* mutants (green) that over-produce glycerol after shifting to 400 mmol l^−1^ sorbitol. The rate shown is the number eggs laid per animal measured every 30 min averaged across 3 animals per plate from 20 independent biological replicates (plates) pooled from two experiments (10 each). (F) Average number of eggs laid by wild-type (black) or *osm-8* (green) mutant animals before (<2 h) and after (>2 h) 2 h of exposure to 400 mmol l^−1^ sorbitol as shown in E. (G) Scatterplot showing the average number of eggs laid within 80 min by wild-type, *tax-2(p694)*, *osm-8(n1518)* or *tax-2; osm-8* double mutant animals incubated on 100 or 400 mmol l^−1^ sorbitol plates. Asterisks indicate significance (****P<0.0001, ***P<0.001, **P<0.01, *P≤0.05; n.s., not significant; two-way ANOVA with Bonferroni correction for multiple comparisons). Error bars represent ±95% confidence intervals for the mean.

To investigate the potential molecular and cellular mechanisms mediating osmolarity-driven regulation of egg-laying behavior, we screened a wide range of genetic mutants. Osmotic avoidance in *C. elegans* is driven by the polymodal ASH neurons that mediate avoidance to mechanosensory and chemical stimuli (Bargmann, 2006; Hart et al., 1999; Kaplan and Horvitz, 1993). These neurons respond and signal through a wide range of molecules including heterotrimeric G proteins including ODR-3 (Roayaie et al., 1998), cyclic nucleotide-gated channels TAX-2/4, and TRPV channels OSM-9 and OCR-2 (Liedtke et al., 2003; Sokolchik et al., 2005; Tobin et al., 2002), among many others (Bargmann, 2006). Previous work has shown that 300 mOsm media fails to inhibit egg laying in *tax-2* or *tax-4* mutants, with inhibition of egg laying in these mutants occurring only at higher (≥375 mOsm) osmolarities (Huang et al., 2023). Consistent with this, we found 400 mmol l^−1^ sorbitol fully inhibited egg laying (>95%) in *tax-2*, *tax-4* and many other sensory defective mutants (Fig. 2A). These results suggest mechanisms necessary for osmotic avoidance are not solely responsible for the inhibition of egg laying by 400 mmol l^−1^ sorbitol. To more completely inhibit chemosensation, we next tested mutants that affect cilia development, required generally for sensory neuron function (Bargmann, 2006; Swoboda et al., 2000) or axonal development (Pujol et al., 2000). As shown in Fig. 2B, these mutants also showed inhibition of egg laying after our 80 min exposure assay, further supporting the idea that sensory perception of high osmolarity is not required for osmotic inhibition of egg laying. For example, we found that 400 mmol l^−1^ sorbitol strongly inhibited (>98%) egg laying in *daf-19* mutants with impaired expression of cilium genes that regulate osmotic avoidance (Swoboda et al., 2000). Conversely, 400 mmol l^−1^ sorbitol inhibited egg laying in *daf-12* mutants only by ∼60% (Fig. 2B). *daf-12* encodes a nuclear-hormone receptor that regulates the expression of genes that control longevity, dauer development, osmotic stress resistance and post-mating shrinking (Antebi et al., 2000; Fisher and Lithgow, 2006; Shi and Murphy, 2014). Interestingly, 400 mmol l^−1^ sorbitol inhibited egg laying almost fully in *daf-19; daf-12* double mutants (>90%), suggesting the osmotic resistance of *daf-12* mutants is mediated by DAF-19-dependent changes in gene expression.

To determine whether the osmotic stress response – in which animals accumulate glycerol to restore water loss – contributed to the inhibition of egg laying, we next tested two mutants, *osm-7* and *osm-8*. These mutants show resistance to osmotic stress via constitutive production of glycerol, preventing the normal loss of hydrostatic pressure that accompanies hyperosmotic stress (Rohlfing et al., 2011; Wheeler and Thomas, 2006). *osm-7* mutants showed only a 40% reduction of egg laying on 400 mmol l^−1^ sorbitol (Fig. 2C; from 1.7 eggs to 1.1 eggs laid after 80 min). We interpret this result in part as being caused by a low baseline level of egg laying in both conditions. *osm-8* mutants maintained high egg laying rates on 100 mmol l^−1^ sorbitol (6.7 eggs) and were inhibited by ∼75% in the high osmolarity condition (1.7 eggs), indicating less inhibition than that seen in wild-type animals (Fig. 2C). To test *osm-8* mutants further, we measured the time course of egg-laying inhibition and recovery after shifting to 400 mmol l^−1^ sorbitol. *osm-8* mutants resumed egg laying within 1 h, significantly earlier than wild-type control animals (Fig. 2E), ultimately laying significantly more eggs within the first 2 h (Fig. 2F). These results suggest that elevation of internal glycerol to pre-emptively maintain a hydrostatic pressure gradient provides resistance to acute shifts to high sorbitol, allowing egg laying. To test whether sensory and osmotic stress function to inhibit egg laying within the same or distinct pathways, we compared egg laying in *tax-2*, *osm-8* and *tax-2; osm-8* double mutants. High osmolarity significantly reduced egg laying in all four strains (Fig. 2G). In wild-type animals, egg laying after 80 min was reduced from ∼15.5 eggs on 100 mmol l^−1^ sorbitol to ∼2.6 eggs on 400 mmol l^−1^ sorbitol, an 83% reduction. In both *tax-2* and *osm-8* single mutants, egg laying was reduced from ∼12.6 or 13.8 eggs on 100 mmol l^−1^ sorbitol to ∼6.0 and 6.4 eggs on 100 mmol l^−1^ sorbitol, a 52% and 53% reduction, respectively. In *tax-2; osm-8* double mutants, egg laying was reduced from ∼7.8 eggs on 100 mmol l^−1^ sorbitol to ∼4.6 eggs on 400 mmol l^−1^ sorbitol, a 42% reduction. While this reduction in osmotic inhibition suggests the sensory and hydrostatic response pathways act in parallel, the overall reduction in egg laying by the *tax-2; osm-8* double mutant and the convergence of all three mutant strains toward ∼5–6 eggs laid on 400 mmol l^−1^ sorbitol complicates such a clear delineation by genetic epistasis.

If high osmolarity inhibits activity in the egg-laying circuit, then mutants with increased Ca^2+^ activity and neurotransmission may overcome this inhibition. To test this hypothesis, we tested hyperactive egg-laying mutants, specifically *goa-1(sa734*) mutants lacking inhibitory Gα_o_­ signaling (Robatzek and Thomas, 2000) or *egl-30(tg26)* gain-of-function mutants with too much excitatory Gα_q_ signaling (Doi and Iwasaki, 2002). Both mutants have higher levels of egg-laying circuit activity and egg-laying behavior on normal osmolarity media (Dhakal et al., 2022; Ravi et al., 2021). While egg laying in these mutants was still reduced ∼70–90% on 400 mmol l^−1^ sorbitol media (Fig. 2D), this was not significantly different from egg laying in the 100 mmol l^−1^ sorbitol condition. *ocr-2(vs29)* mutant animals with altered TRPV channel function and hyperactive egg laying (Jose et al., 2007) were similarly inhibited by high osmolarity media (Fig. 2D). Like the osmotic-resistant mutants, these hyperactive egg-laying behavior mutants have reduced brood sizes (Bastiani et al., 2003; Ségalat et al., 1995), so it is hard to conclude that the effects of high osmolarity are solely due to cessation of circuit activity versus additional effects on egg production (see Fig. 1G). Overall, these results suggest that sensory signaling is not strictly required for cessation of egg laying in response to hyperosmotic media exposure. This is supported by the wide range of mutants, defective in sensory neuron development or signaling, that still show inhibition by 400 mmol l^−1^ sorbitol exposure. Our results from osmotic-resistant glycerol accumulation mutants suggest instead that physiological mechanisms that restore a hydrostatic pressure gradient allow egg laying after shifts to high osmolarity. Together, these results support a model where high osmolarity activates sensory neurons that signal to inhibit egg laying while also causing biophysical effects that include disruption of a hydrostatic pressure gradient that facilitates egg release.

### Recovery from hyperosmotic stress induces an acute increase in egg laying

**Fig. 3.**
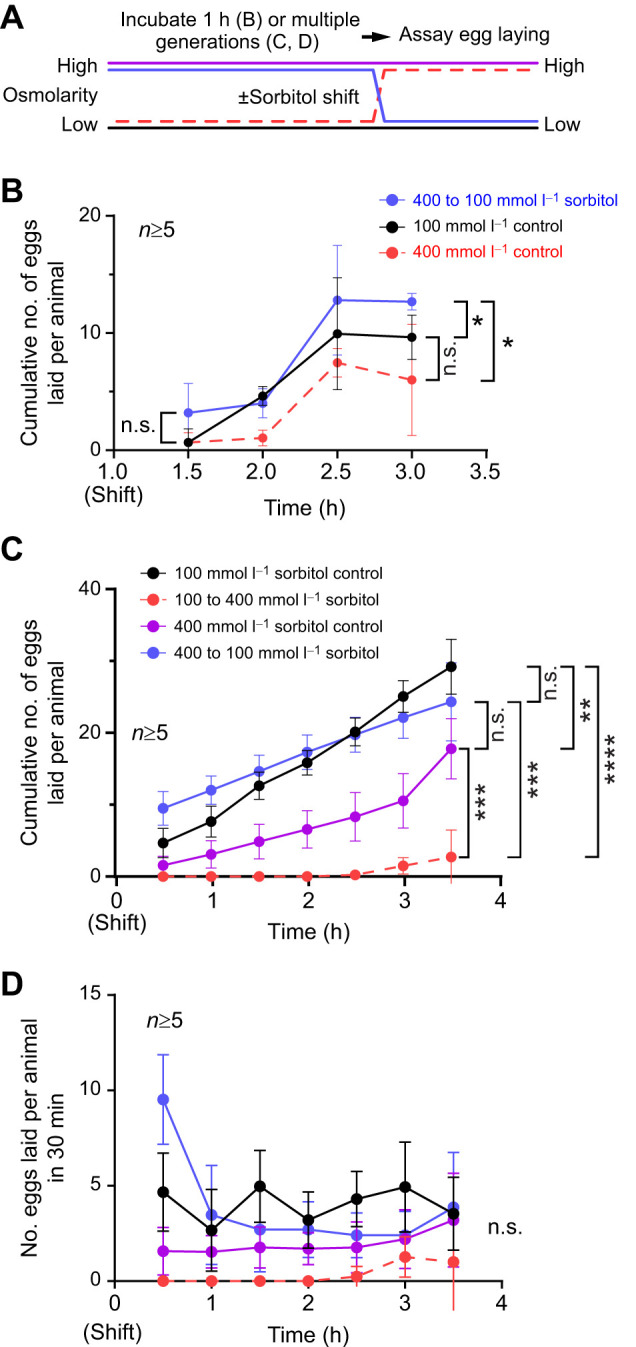
**Rescue from hyperosmotic conditions induces a homeostatic increase in egg laying.** (A) Diagram of the experimental setup. Animals were incubated for either 1 h on agar–sorbitol plates (B) or for multiple generations on NGM agar plates containing 100 or 400 mmol l^−1^ sorbitol (C,D; see Materials and Methods). Thereafter, animals were moved to either 100 or 400 mmol l^−1^ agar–sorbitol plates (B) or NGM agar–sorbitol plates (C,D) and the number of eggs laid on the new plates was counted every 30 min. (B) Cumulative number of eggs laid by animals incubated for 1 h, or their entire lives (C), followed a ‘shift’ to the indicated treatment plates (denoted by color). (D) The number of eggs laid per each 30 min interval from data in C. All dots in B–D represent the average response from multiple animals within a plate. The egg-laying response from a plate was divided by the number of animals on the plate to obtain the average response per animal. Error bars represent ±95% confidence interval. A mixed model statistical analysis with a Bonferroni correction for multiple comparisons was performed for each time series data. Because of the large number of comparisons, only significance at the beginning and end of each time series is shown; only significance for the last time point is shown in C and D (****P<0.0001, ***P<0.001, **P<0.01, *P<0.05; n.s., not significant).

We have previously shown that the egg-laying circuit induces a homeostatic increase in egg laying and in Ca^2+^ activity of the HSNs after artificial accumulation of unlaid eggs (Ravi et al., 2018b). As hyperosmotic inhibition of egg laying leads to an increase in egg retention (Fig. 1G), we next questioned whether animals incubated in high-osmolarity conditions would show an increase in egg-laying behavior once they were moved back to 100 mmol l^−1^ sorbitol. As shown in Fig. 3A, two control conditions were created: the 100 mmol l^−1^ (low) sorbitol control from which animals were moved to a different set of 100 mmol l^−1^ sorbitol plates, and the 400 mmol l^−1^ (high) sorbitol control condition from which animals were similarly moved from one set of 400 mmol l^−1^ sorbitol plates to a different set. One experimental condition (400 to 100 mmol l^−1^ sorbitol) involved moving animals incubated on 400 mmol l^−1^ sorbitol for 1 h to 100 mmol l^−1^ sorbitol plates (Fig. 3A). Consistent with prior results (Fig. 1E), 100 mmol l^−1^ sorbitol control animals showed a steady rate of egg laying despite the transfer while 100 mmol l^−1^ sorbitol animals transferred to 400 mmol l^−1^ sorbitol showed general inhibition of egg laying followed by resumption after 2.5 h of exposure to hyperosmotic stress (Fig. 3B). Animals that were switched from 400 to 100 mmol l^−1^ sorbitol plates laid more eggs over time, ∼12 eggs on average per worm over 2 h, compared with the ∼5–10 eggs from either group (Fig. 3B). This result is consistent with a homeostatic rebound effect where an elevated ‘high inside’ hydrostatic pressure gradient is more favorable for egg laying immediately following the osmotic shift.

As the maximal production of glycerol from acclimatization to osmotic stress takes ∼24 h (Lamitina et al., 2004), we next tested whether animals incubated on high sorbitol plates over multiple generations would show similar responses. Because these experiments require long-term incubations and growth that might not be optimal on simple agar plates, we grew worms on standard NGM agar supplemented with either 100 or 400 mmol l^−1^ sorbitol. If acclimatization to hyperosmotic environments is sufficient to prevent inhibition of egg laying, then we would expect no difference between animals grown on NGM media containing 100 or 400 mmol l^−1^ sorbitol for their whole lives. We did find that animals shifted from NGM agar plates containing 400 mmol l^−1^ sorbitol to the same media showed modestly lower levels of egg laying, reaching an average of ∼20 eggs laid per animal over 3.5 h, compared with the ∼25 eggs laid per worm for the NGM plus 100 mmol l^−1^ sorbitol control group (Fig. 3C). This is significantly greater than the ∼3 eggs laid by animals shifted from NGM media with 100 mmol l^−1^ sorbitol to NGM media plus 400 mmol l^−1^ sorbitol. In contrast, animals grown on high sorbitol and down-shifted to 100 mmol l^−1^ sorbitol showed a significant increase in egg laying for the first 30 min before declining to a similar steady-state level seen in 100 or 400 mmol l^−1^ sorbitol-treated control animals (Fig. 3D). Overall, these results show that recovery from hyperosmotic stress induces an acute rebound in egg laying (<30 min), consistent with a high-inside pressure gradient promoting egg release, followed by a return to normal rates of egg laying once the pressure gradient returns to normal. The low egg-laying rates maintained by animals kept on high osmolarity over multiple generations suggests a chronic inhibition of egg laying and/or germline activity that reduces egg laying even after the hydrostatic pressure gradient is restored.

### Vulval muscle Ca^2+^ activity does not recover following chronic hyperosmotic exposure

**Fig. 4.**
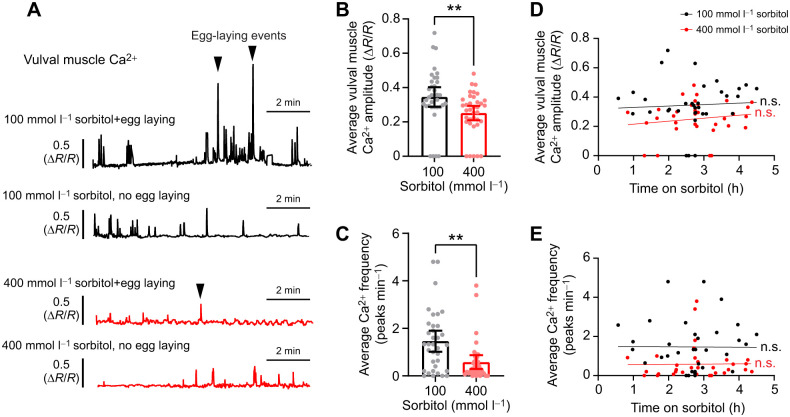
**Vulval muscle Ca^2+^ activity remains inhibited after chronic exposure to high osmolarity.** (A) Representative GCaMP5/mCherry fluorescence traces of animals exposed to NGM agar plates containing either 100 mmol l^−1^ (black) or 400 mmol l^−1^ (red) sorbitol. Traces show examples of animals in which no egg laying or at least one egg laying event (denoted by arrowhead) was observed. (B) Average vulval muscle Ca^2+^ response from individual animals from both sorbitol conditions. Asterisks indicate significance (**P<0.01; Mann–Whitney test). (C) Average Ca^2+^ frequency from both sets of animals (**P<0.01; Mann–Whitney test). (D,E) Scatterplot with linear regression showing no correlation between average vulval muscle Ca^2+^ amplitude (D) or average Ca^2+^ frequency (E) and time exposed to sorbitol. n.s., no significance (simple linear regression).

Acute exposure to high osmolarity has been shown to cause a decrease in HSN, ventral C (VC) neuron, and vulval muscle Ca^2+^ peak frequency (Zhang et al., 2008, 2010). If *C. elegans* can resume egg-laying behavior after chronic exposure to hyperosmotic environments, then presumably the egg-laying circuit shows resumption of activity. To test this, we recorded Ca^2+^ activity from the vulval muscles of animals incubated on 100 or 400 mmol l^−1^ sorbitol plates for different amounts of time. Vulval muscle Ca^2+^ activity and egg-laying events were observed in both conditions (Fig. 4A). However, animals on 100 mmol l^−1^ plates laid eggs in 23% of recordings (8/35) while animals on 400 mmol l^−1^ plates laid eggs in only 3% of all recordings (1/36), a significant difference (*P*=0.0333; Fisher's exact test). Consistent with these lower rates of egg laying, hyperosmotic treatment yielded a decrease in average vulval muscle Ca^2+^ transient peak amplitude and frequency on a per animal basis (Fig. 4B,C). Contrary to the observed resumption of egg-laying behavior (Fig. 1), both vulval muscle Ca^2+^ transient amplitude and peak frequency did not show a positive correlation with time spent on high osmolarity plates (Fig. 4D,E). Together, these data suggest that high osmolarity depresses vulval muscle Ca^2+^ activity while in a hyperosmotic environment, likely until they fully adapt. Accumulation of eggs may then serve to promote egg-laying behavior once animals reach a critical point of egg accumulation, as noted previously (Collins et al., 2016; Medrano and Collins, 2023).

### Hyperosmotic inhibition of egg laying is mediated in part by physical deficiencies in egg release

**Fig. 5.**
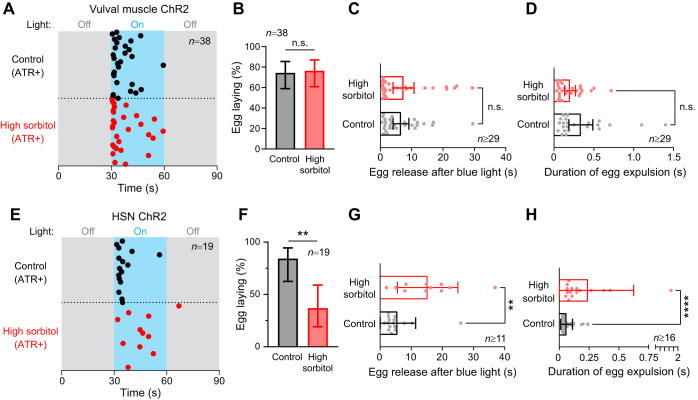
**Optogenetic induction of egg laying causes delayed egg-laying responses from animals exposed to high osmolarity.** (A) Egg-laying pattern following optogenetic activation of the vulval muscles in transgenic animals expressing Channelrhodopsin-2 (ChR2). Dots indicate the timing of the first egg-laying event. Gray shaded regions represent blue light off while the blue shaded region represents blue light on. ATR, all-trans retinal. (B) Percentage of animals showing egg laying following optogenetic stimulation of the vulval muscles (n.s., not significant; Fisher's exact test). (C) Bar graph with scatterplot showing the mean time taken for the first egg-laying event since blue light activation of the muscles (n.s., not significant; Mann–Whitney test). (D) Bar graph with scatterplot showing the mean time taken for eggs to be expelled from the uterus to the outside environment (n.s., not significant; Mann–Whitney test). (E) Egg-laying pattern following optogenetic activation of the HSN neurons in transgenic animals expressing ChR2 (as in A). (F) Percentage of animals showing egg laying following optogenetic stimulation of the HSNs. Asterisks indicate significance (**P=0.0069; Fisher's exact test). (G) Bar graph with scatterplot showing the mean time taken for egg-laying events after blue light activation of the HSNs (**P=0.0022; Mann–Whitney test). (H) Bar graph with scatterplot showing the mean time taken for egg expulsion during an egg-laying event after blue light activation of the HSNs (****P<0.0001; Mann–Whitney test). Error bars represent ±95% confidence interval for the mean.

If hyperosmotic inhibition of egg laying is mediated by depression of circuit activity or a disruption of a pressure gradient required for proper expulsion of eggs, then animals forced to lay eggs in hyperosmotic conditions may show physical deficiencies depending on the way egg laying is induced. To test this hypothesis, we induced egg laying via optogenetic stimulation in transgenic animals expressing ChR2 in the vulval muscles to bypass ongoing egg-laying circuit activity (Kopchock et al., 2021; Yan et al., 2025). Continuous blue-light exposure for 30 s led to egg laying in animals on control or high sorbitol plates (Fig. 5A), with ∼80% of animals in both groups showing light-induced egg laying (Fig. 5B). We observed no deficiency in the amount of time taken for the first egg to be released (Fig. 5C) or in the time required for eggs to be fully expelled from the uterus following optogenetic induction of vulval activity (Fig. 5D). These results indicate that acute changes in the hydrostatic pressure gradient do not significantly alter egg release when driven in response to sustained optogenetic vulval muscle contraction.

We repeated the experiment using a transgenic strain expressing ChR2 in the HSNs (Emtage et al., 2012; Gürel et al., 2012). This strain shows robust light-induced egg laying on NGM control plates (Fig. 5E), but fewer of the animals showed egg release (∼40%) in response to continuous blue-light stimulation on high sorbitol (Fig. 5F). Surprisingly, on high sorbitol plates, there was a delay in the onset of egg release, with the first egg being laid after 10 s compared with ∼4 s for animals on NGM control plates (Fig. 5G). We also found that each egg physically took longer to be released on hyperosmotic media (Fig. 5H), with egg passage occurring over 250 ms on high sorbitol plates rather than the 50 ms on NGM control plates, suggesting that the kinetics of egg release is sensitive to the hydrostatic pressure gradient. Together, these results suggest that hyperosmotic inhibition may prevent the normal activation of the vulval muscles for egg laying and/or efficient egg release. High sorbitol reveals deficiencies in either the optogenetic activation of the HSNs themselves or the downstream responses of the vulval muscles that drive egg laying.

**Fig. 6.**
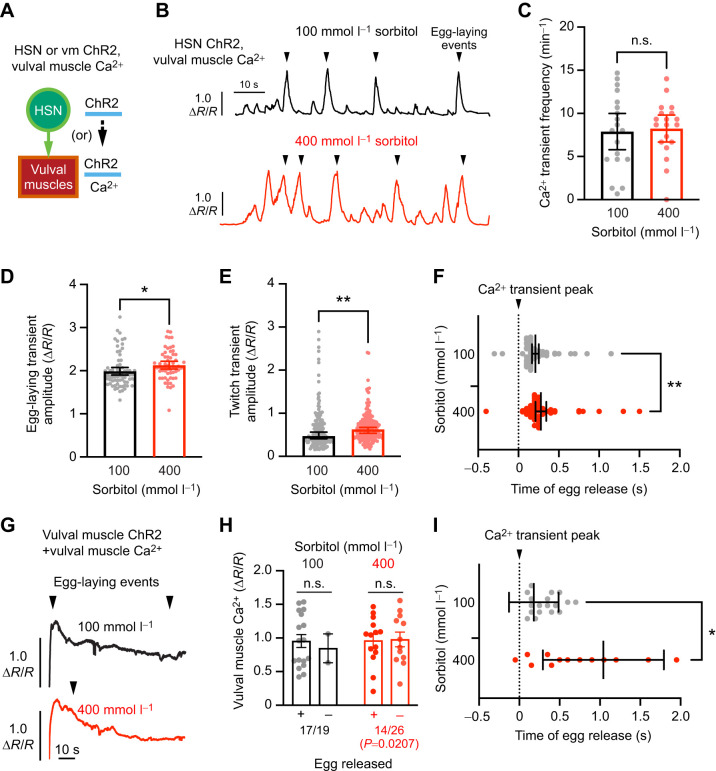
**High osmolarity slows egg release following vulval muscle contraction.** (A) Diagram of the experiment. ChR2 is transgenically expressed either in the HSNs or vulval muscles for optogenetic stimulation and GCaMP5 and mCherry are expressed in the vulval muscles for ratiometric Ca^2+^ imaging following a ∼1 h shift to agar plates containing 100 or 400 mmol l^−1^ sorbitol. (B) Representative vulval muscle Ca^2+^ traces following optogenetic HSN stimulation. Egg-laying events are indicated by arrowheads. (C) Average frequency of vulval muscle Ca^2+^ transients following optogenetic stimulation. Dots indicate the per recording average with *n*=19 animals measured per condition. Error bars indicate ±95% confidence intervals for the mean (n.s., not significant; Student's *t*-test). (D,E) Average vulval muscle Ca^2+^ transient amplitudes following HSN optogenetic activation during egg laying (D) and twitch (e.g. non-egg laying, E) events. Dots indicate single transient amplitudes pooled from 19 animals per condition. Asterisks indicate significance (in D *P=0.0285, Student's *t*-test; in E **P=0.0034, Mann–Whitney test). (F) Lag time between vulval muscle Ca^2+^ transient peak (dotted line) and egg release. Dots indicate timing of individual egg-laying events pooled from 19 animals. Error bars indicate ±95% confidence intervals for the mean (**P=0.0067; Mann–Whitney test). (G) Representative vulval muscle GCaMP5/mCherry fluorescence traces of Ca^2+^ activity following optogenetic vulval muscle stimulation. Egg-laying events are indicated by arrowheads. (H) Vulval muscle Ca^2+^ transient amplitudes following vulval muscle optogenetic stimulation. Dots indicate Ca^2+^ transient amplitudes immediately prior to egg release (plus signs). For those traces where no egg laying was observed (minus signs), dots indicate the peak Ca^2+^ transient amplitude from each recording. Error bars indicate standard error for the mean (n.s., not significant; one-way ANOVA with Bonferroni correction for multiple comparisons). The fraction of animals showing egg-laying events following optogenetic stimulation was significantly different (*P*=0.0207, Fisher exact test; 100 mmol l^−1^ sorbitol: *n*=19 animals, 400 mmol l^−1^ sorbitol: *n*=26 animals). (I) Lag time between vulval muscle Ca^2+^ transient peak (dotted line) and egg release. Dots indicate the timing of individual egg-laying events relative to each Ca^2+^ transient peak. Error bars indicate ±95% confidence intervals for the mean (*P=0.0156; Mann–Whitney test).

To examine directly how changes in the osmotic pressure gradient affect vulval muscle activity and/or egg release, we measured vulval muscle Ca^2+^ following optogenetic stimulation of the HSNs or vulval muscles (Fig. 6A). We moved animals from NGM agar to plates supplemented with either 100 or 400 mmol l^−1^ sorbitol for at least 1 h prior to imaging, with all animals incubated on low or high osmolarity media for ≤3 h. Under these conditions, optogenetic stimulation of the HSNs in animals on either 100 or 400 mmol l^−1^ sorbitol caused a robust induction of vulval muscle Ca^2+^ transients and egg laying (≥85% of animals) during a 90 s recording period (Fig. 6B). We observed no significant differences in the frequency of induced vulval muscle Ca^2+^ transients or egg laying in animals treated with 400 mmol l^−1^ sorbitol (Fig. 6C), although we did observe a significant increase in the amplitude of Ca^2+^ transients during both egg-laying (Fig. 6D) and twitch transients (Fig. 6E). This suggests either that high osmolarity increases the vulval muscle Ca^2+^ response or that increased Ca^2+^ responses are necessary to drive vulval muscle contraction following osmotic acclimation. Our previous HSN ChR2 experiments showed high osmolarity inhibited egg release – a result not observed in these Ca^2+^ imaging experiments. The source of this discrepancy is not clear, although it may relate to differences in media composition or how the HSNs are optogenetically stimulated, with pulsed illumination being used for GCaMP5 imaging rather than the continuous blue light exposure typically used in behavior experiments.

We have previously shown that eggs are released immediately after the vulval muscles reach their peak Ca^2+^ activity (Collins et al., 2016; Medrano and Collins, 2023). To measure whether high osmolarity affects the coordination of vulval muscle Ca^2+^ activity and egg release, we measured how long it took for each egg to be released after the vulval muscle Ca^2+^ transient peak was observed. As shown in Fig. 6F, in animals grown on 100 mmol l^−1^ sorbitol, this lag between Ca^2+^ peak and egg release was ∼0.2 s on average (median 0.15 s). Animals on 400 mmol l^−1^ sorbitol showed a significant delay to ∼0.28 s (median 0.2 s) between vulval muscle Ca^2+^ peak and egg laying. While this increased lag on 400 mmol l^−1^ sorbitol represents a delay of only 1 frame (0.05 s), it is statistically significant (*P*=0.0067, Mann–Whitney test). Together, these results suggest changes in osmolarity affect the hydrostatic pressure gradient and the timing of egg ejection following vulval muscle contraction.

We separately compared how changes in osmolarity affected vulval muscle Ca^2+^ activity and egg laying following direct optogenetic stimulation. As shown previously (Kopchock et al., 2021), optogenetic stimulation of the vulval muscles drives an immediate and robust vulval muscle Ca^2+^ activity and egg laying (Fig. 6G). We found no significant difference in the pattern or peak amplitude of vulval muscle Ca^2+^ responses in animals on 100 mmol l^−1^ or 400 mmol l^−1^ sorbitol (Fig. 6H). This was surprising because animals on 400 mmol l^−1^ sorbitol laid eggs only half as frequently as those on 100 mmol l^−1^ sorbitol (Fig. 6H), similar to that seen previously following optogenetic stimulation of the HSNs (Fig. 5F). This led to a significant increase in the lag observed between the vulval muscle Ca^2+^ peak and egg release (Fig. 6I). In animals grown on 100 mmol l^−1^ sorbitol, the lag between Ca^2+^ peak and egg release was ∼0.18 s on average (median 0.25 s), similar to that seen following HSN optogenetic stimulation (compare Fig. 6F and I). However, animals on 400 mmol l^−1^ sorbitol showed a significant delay to ∼1 s (median 0.675 s) between vulval muscle Ca^2+^ peak and egg laying following optogenetic vulval muscle stimulation (Fig. 6I). These results again suggest vulval muscle contraction is insufficient for egg release in animals where the hydrostatic pressure has been disrupted by high osmolarity. Egg laying therefore has two components: first, vulval muscle Ca^2+^ activity drives contraction and vulval opening; second, a high-inside hydrostatic pressure gradient powers egg release into the environment.

## DISCUSSION

In this study, we investigated roles for both sensory and biophysical pathways in the inhibition of *C. elegans* egg-laying behavior by high osmolarity. Chemosensory mutants with defects in the detection of high osmolarity continued to lay eggs in high sorbitol, suggesting the well-understood sensory pathway mediating osmotic avoidance is not mediating the egg-laying inhibition. However, mutants that produce excessive glycerol – which would maintain the animals' hydrostatic pressure gradient – showed increased egg laying, resuming egg laying more quickly upon acute shifts to hyperosmolarity. Egg laying in animals cultured under chronic hyperosmotic conditions also showed resistance. Intriguingly, such animals showed an acute increase in egg laying when shifted back to hypo-osmotic conditions, consistent with the idea that the hydrostatic pressure gradient, not long-term adaptive changes, underlies the egg-laying responses. Resumption of egg laying after chronic exposure to high osmolarity does not quite restore the normal rate of vulval muscle Ca^2+^ activity nor egg laying, possibly because of additional long-term effects on egg production overall (Fig. 1G, right). Previous work has identified genetic mechanisms that control egg laying that may allow progeny survival in environments with different kinds of stress (Mignerot et al., 2024). We found optogenetic stimulation of either the HSNs or the vulval muscles was sufficient to induce vulval muscle Ca^2+^ activity and egg release even when egg laying was otherwise inhibited by high osmolarity conditions. Interestingly, high osmolarity slowed egg release following vulval muscle Ca^2+^ activity. Taken together, these results suggest that hyperosmotic environments can inhibit egg laying via activation of a sensory pathway and via a biophysical pathway.

Support for the idea that high osmolarity can inhibit egg laying via two distinct pathways is bolstered by recent results showing that increasing the concentration of sorbitol inhibits egg laying (Huang et al., 2023). These authors found that animals with mutations in *tax-2* or *tax-4* can lay eggs in 300 mOsm conditions, while wild-type animals are unable to. Interestingly, these same mutants are unable to lay eggs at higher concentrations (375 or 450 mOsm), as we observed (at 400 mOsm), suggesting egg laying is inhibited even when those sensory pathways are genetically disrupted. Such a biophysical mechanism is consistent with our results showing *osm-8* mutants which have elevated glycerol show more egg laying on 400 mmol l^−1^ sorbitol. Separate pathways in the detection of a physical stimulus have been observed in the *C. elegans* gentle and harsh touch response where different mechanosensors and mechanosensory neurons are responsible for detecting and conveying information on the physical stimulus depending on the intensity of that stimulus (Chalfie, 2014; Fechner and Goodman, 2018; Goodman, 2006). In this case, egg laying is physically inhibited by a non-permissive osmotic pressure barrier that regulates a hydrostatic pressure gradient across the cuticle that supports egg release upon vulval opening. Such a model would predict a physical limit at which the osmotic pressure becomes too great for egg laying to occur. In support of this, we observed that animals acclimated to or maintained on 400 mmol l^−1^ sorbitol over multiple generations show a steady, but lower, level of egg laying when compared with animals maintained in 100 mmol l^−1^ sorbitol media (Figs 1 and 3). This suggests that egg release occurs only when a sufficient buildup of pressure, perhaps through increased egg accumulation (Fig. 1G), is present to overcome physical limitations and/or the inhibition of circuit activity that accompanies osmosensory input. Future studies looking at whether acute increases in internal pressure, such as microinjection (Medrano and Collins, 2023), can bypass osmotic inhibition of egg laying may provide further evidence for the biophysical blocking of egg laying model.

A prediction of our biophysical model is that hyperosmotic inhibition of egg laying acts independent of egg-laying circuit activity. In other words, animals on high osmolarity plates would show normal levels of Ca^2+^ activity in the egg laying circuit but would simply be unable to physically lay eggs. Although we observed a steady (albeit reduced) rate of egg laying from animals acclimated and maintained at high osmolarity (Fig. 3C,D), vulval muscle Ca^2+^ activity was reduced in animals exposed to 400 mmol l^−1^ sorbitol for up to 4.5 h. (Fig. 4D,E). This supports past work that suggests that sensory signaling of high osmolarity environments signals to inhibit egg-laying activity (Huang et al., 2007; Zhang et al., 2008). However, what happens to the activity of the egg-laying circuit (the HSNs, VCs, uterine-vulva 1 cells (uv1s) and vulval muscles) after long-term growth on high osmolarity media remains unclear. We hypothesize a return of the same pattern of two-state circuit activity that accompanies the onset of the egg-laying active state in normal osmotic conditions (Collins et al., 2016; Ravi et al., 2018b; Waggoner et al., 1998; Olson et al., 2023). Thus, future studies should focus on investigating the long-term changes in the patterns of Ca^2+^ activity of animals chronically exposed to hyperosmotic environments. The use of sensory mutants should allow direct tests of the distinct contributions and mechanisms of the two pathways of hyperosmotic inhibition (Zhang et al., 2022). Alternatively, high osmolarity may also drive changes in circuit morphology, synaptic structure or connections that underlie the acute inhibition we observed. While we did not see any dramatic alterations in vulval muscle morphology (data not shown), our previous work has shown that acute microinjection can lead to altered egg positioning around the vulva (Medrano and Collins, 2023). Other types of mechanical stimulation activate the uv1 cells, whose signaling can inhibit egg laying (Collins et al., 2016; Banerjee et al., 2017; Yan et al., 2025). High osmolarity may also drive changes in synaptic structure or connectivity that could in the future be measured (Feinberg et al., 2008). We would predict that any such induced changes would then be restored coincident with the resumption of egg laying.

Upon movement from hyperosmotic to isotonic environments (now relatively hypoosmotic to hyperosmotic-acclimated animals), *C. elegans* shows a rapid efflux of glycerol, losing about 25% of accumulated glycerol within a few hours (Lamitina et al., 2004). This efflux is likely triggered via swelling and mediated by the glycerol-permeable aquaglyceroporins AQP-1 and AQP-7 (Huang et al., 2007; Lamitina et al., 2004). As such, it is possible that *osm-7* and *osm-8* mutants have increased baseline levels of glycerol excretion to acclimate to relatively hypotonic environments, which would typically be isotonic to animals lacking such mutations. A rapid excretion of glycerol also helps explain our observations that moving animals from high osmolarity to a relatively lower osmolarity induces an increase in egg laying that occurs only within the first 30 min. This is because, similarly, shifting animals from high to lower osmolarity causes an acute initial increase in relative body size, followed by a return to a typical size that occurs within 20 min and is likely mediated by glycerol or water excretion (Huang et al., 2007; Lamitina et al., 2004). Consistent with a model in which an acute increase in internal pressure induces egg laying, the sudden influx of water would then lead to an increase in hydrostatic pressure and egg-laying behavior, as we observed (Fig. 3). Past work has shown that acute shifts to ‘low’ external osmolarity promote egg-laying circuit activity (Zhang et al., 2008), and our results would predict that any shift from a relatively higher to lower osmolarity would increase the steepness of the ‘high inside’ hydrostatic pressure gradient, inducing egg-laying circuit activity and behavior. Indeed, this interpretation is consistent with our results showing artificial egg accumulation or even acute injection drives a robust activation of egg-laying circuit activity and behavior (Medrano and Collins, 2023). Thus, future studies should focus on investigating whether induction of egg-laying circuit activity is due to a signaling pathway or to the influx of water causing an acute stretch (or both). Measurements of circuit activity during these acute shifts would also help reveal the mechanisms by which egg laying is activated or inhibited.
